# Multicenter Study of Susceptibility of Aspergillus Species Isolated from Iranian University Hospitals to Seven Antifungal Agents

**DOI:** 10.1128/spectrum.02539-21

**Published:** 2022-05-17

**Authors:** Parisa Badiee, Teun Boekhout, Ali Zarei Mahmoudabadi, Rasoul Mohammadi, Seyyed Amin Ayatollahi Mousavi, Mohammad Javad Najafzadeh, Jafar Soltani, Jamal Hashemi, Kambiz Diba, Abdolkarim Ghadimi-Moghadam, Ali Reza Salimi-Khorashad, Tahereh Shokohi, Maneli Amin Shahidi, Fatemeh Ghasemi, Hadis Jafarian

**Affiliations:** a Clinical Microbiology Research Center, Shiraz University of Medical Sciences, Shiraz, Iran; b Westerdijk Fungal Biodiversity Institute, Utrecht, The Netherlands; c Institute for Biodiversity and Ecosystem Dynamics, IBED, University of Amsterdam, Amsterdam, The Netherlands; d Infectious and Tropical Diseases Research Center, Health Research Institute, Ahvaz Jundishapur University of Medical Sciences, Ahvaz, Iran; e Department of Medical Parasitology and Mycology, School of Medicine, Infectious Diseases and Tropical Medicine Research Center, Isfahan University of Medical Sciences, Isfahan, Iran; f Department of Medical Parasitology and Mycology, Faculty of Medicine, Kerman University of Medical Sciences, Kerman, Iran; g Department of Medical Parasitology and Mycology, Mashhad University of Medical Sciences, Mashhad, Iran; h Department of Pediatrics, Faculty of Medicine, Kurdistan University of Medical Sciences, Sanandaj, Iran; i Medical Mycology Department, School of Public Health Research, Tehran University of Medical Sciences, Tehran, Iran; j Cellular and Molecular Research Center, Urmia University of Medical Sciences, Urmia, Iran; k Department of Pediatrics Infectious Disease, Emmam Sajjad Hospital, Yasuj University of Medical Sciences, Yasuj, Iran; l Department of Parasitology and Mycology, School of Medicine, Infectious Diseases and Tropical Medicine Research Center, Zahedan University of Medical Sciences, Zahedan, Iran; m Department of Medical Mycology, School of Medicine, Invasive Fungi Research Centre (IFRC), Communicable Diseases Institute, Mazandaran University of Medical Sciences, Sari, Iran; Mycology Laboratory, Wadsworth Center

**Keywords:** amphotericin B, antifungal susceptibility, *Aspergillus*, azoles, echinocandins, Iran

## Abstract

Aspergillus species are a major cause of life-threatening invasive infections and noninvasive diseases. This study seeks to investigate the frequency of Aspergillus species among Iranian patients and their susceptibility to seven antifungals. In a cross-sectional study, 233 Aspergillus isolates were collected from 11 university hospitals in Iran between 2018 and 2021. Aspergillus isolates were identified based on colony morphology, microscopic characteristics, PCR-restriction fragment length polymorphism (RFLP), and sequencing of the beta-tubulin gene. The CLSI M38-A2 reference methodology was used for antifungal susceptibility testing of amphotericin B, voriconazole, posaconazole, itraconazole, luliconazole, isavuconazole, and caspofungin. Members of Aspergillus section *Flavi* (117/233, 50.2%), Aspergillus section *Nigri* (77/233, 33.1%), Aspergillus section *Fumigati* (21/233, 9%), Aspergillus section *Terrei* (14/233, 6%), Aspergillus pseudodeflectus (2/233, 0.85%), and Aspergillus melleus (2/233, 0.85%) were isolated from the samples. The lowest 0.25 MIC_90_ values for all isolates tested were for luliconazole (0.016 μg/mL) and isavuconazole (0.250 μg/mL), and the highest value was observed for itraconazole (≥ 8μg/mL). The 90% minimum effective concentration (MEC_90_) value for caspofungin was 0.125 μg/mL. MIC_90_ values for voriconazole, amphotericin B, and posaconazole were 1, 2, and 2 μg/mL, respectively. The non-wild-type species were presented for amphotericin B (3%), voriconazole (1.3%), posaconazole (2.6%), luliconazole (1.3%), isavuconazole (1.7%), and caspofungin (4.7%). Positive correlations in the MIC values of azole antifungals were observed, and using one azole increases the MIC value rates of other ones. None of the species were pan-azole resistant. Species of Aspergillus section *Flavi* were the most common Aspergillus species isolated from Iranian samples. Luliconazole, caspofungin, and isavuconazole present the most effective antifungal agents for treatment of infection due to Aspergillus species. Susceptibility tests should be performed frequently in each region for the best management of patients.

**IMPORTANCE** Aspergillus species are the leading cause of invasive aspergillosis in immunocompromised hosts. The susceptibility of Aspergillus species to antifungal agents might be different. Azole-resistant species have emerged worldwide. Performing susceptibility testing in each region can help in the best management of patients. Here, we show the epidemiology and distribution of Aspergillus species in Iran and their susceptibility patterns for seven antifungal agents. The significant points of the present study are that species of Aspergillus section *Flavi* are the most prevalent Aspergillus species isolated from 11 university hospitals. Luliconazole, caspofungin, and isavuconazole were effective antifungal agents against all Aspergillus species.

## INTRODUCTION

Aspergillus species cause human infections usually referred to as aspergillosis and comprise, e.g., otomycosis, pulmonary infections, and systemic life-threatening invasive infections (1). Invasive infections occur in immunocompromised patients such as those suffering from hematologic diseases, transplant recipients (solid organs and bone marrow), patients receiving corticosteroids, and patients after viral infections such as COVID-19 (2–6). According to guidelines from the European Society of Clinical Microbiology and Infectious Diseases (ESCMID), voriconazole (VOR) and isavuconazole (ISA) are the drugs of choice for the treatment of Aspergillus infections (7). Many Aspergillus species are phylogenetically closely related species belonging to species complexes. The most clinically relevant species causing aspergillosis include species of Aspergillus section *Flavi*, Aspergillus section *Fumigati*, Aspergillus section *Terrei*, and Aspergillus section *Nigri*. Characterization of Aspergillus epidemiology due to resistance to antifungal agents is clinically important. There are many reports of Aspergillus infections caused by azole-resistant isolates. Antifungal resistance in Aspergillus species plays a critical role in the management of aspergillosis in immunocompromised patients. Because of the extensive use of environmental azole fungicides, e.g., preservatives in the food industry and azole-based fungicides in agriculture, and the use of antifungals in hospital wards, azole resistance is on the rise worldwide (8). In the Netherlands, the etiologic agents of 11.3% of patients with invasive aspergillosis were azole-resistant isolates with a mortality rate of 50 to 100% (9, 10). Azole resistance was reported in many Aspergillus species from Aspergillus section *Fumigati*, Aspergillus section *Flavi*, and Aspergillus section *Terrei* (11). The infection rates of azole-resistant isolates of Aspergillus section *Fumigati* species were reported to be 28.1% in the Netherlands, 7.9% in Taiwan, and 6.6% in Pakistan (12–14). The mortality rates are higher in patients infected by amphotericin B (AMB)- or azole-resistant isolates than in those infected by susceptible ones. Antifungal susceptibility testing may help to decide on proper treatment of patients with suspected invasive aspergillosis. Unfortunately, due to limited samples, antifungal susceptibility testing of fungi is not routinely done in most clinical laboratories, and there is some variability in the results of testing among laboratories. In this study, the epidemiological agents were identified and the susceptibility patterns of Aspergillus isolates from 11 university hospitals in Iran to seven antifungal agents were evaluated using Clinical and Laboratory Standards Institute (CLSI) M38-A2, M61, and M57 (15–17).

## RESULTS

A total of 233 Aspergillus isolates were collected from 11 Iranian university hospitals, consisting of 191 clinical isolates and 42 obtained from hospital wards.

**FIG 1:**
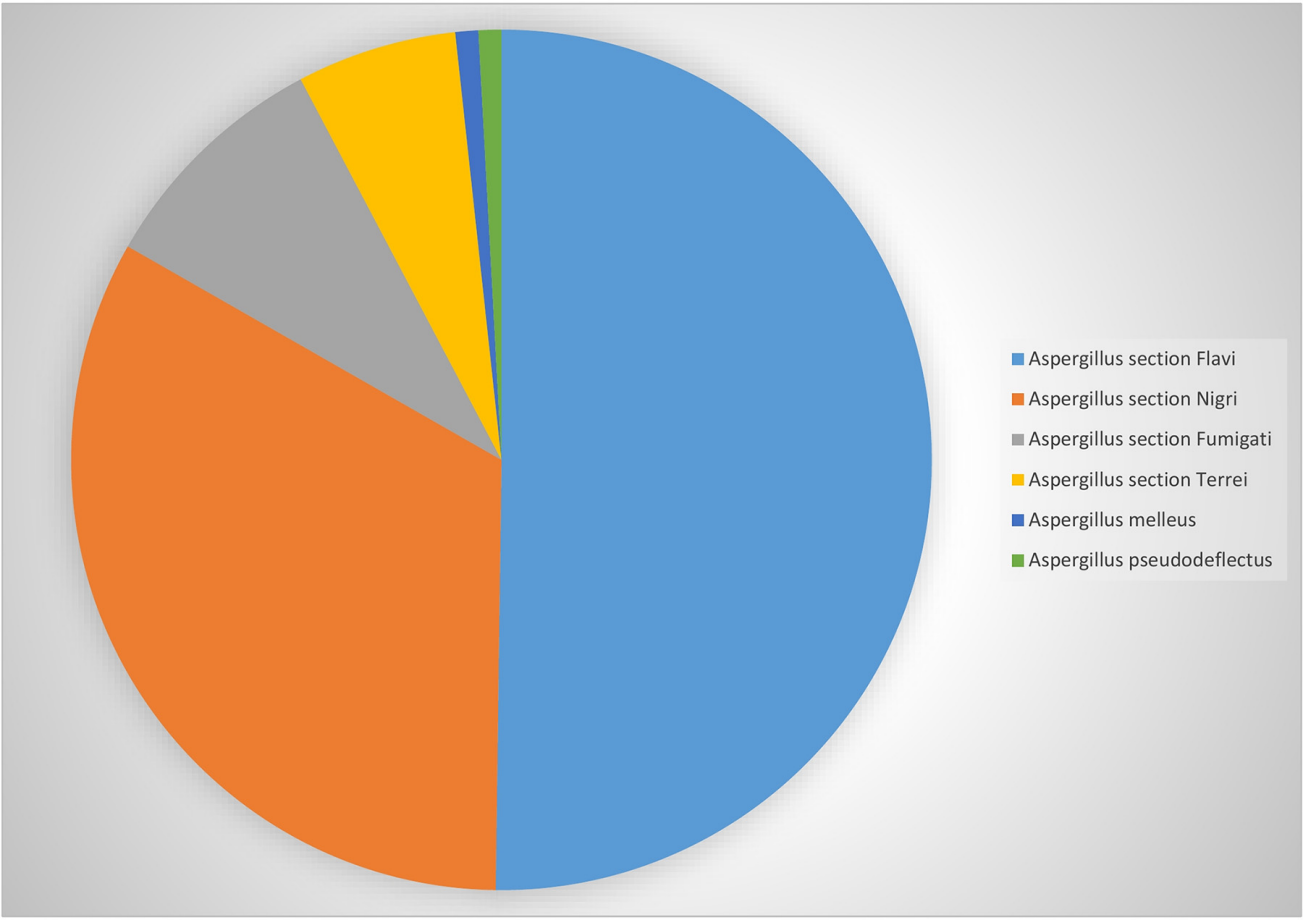
Aspergillus species isolated from clinical samples in 11 university hospitals in Iran.

**FIG 2:**
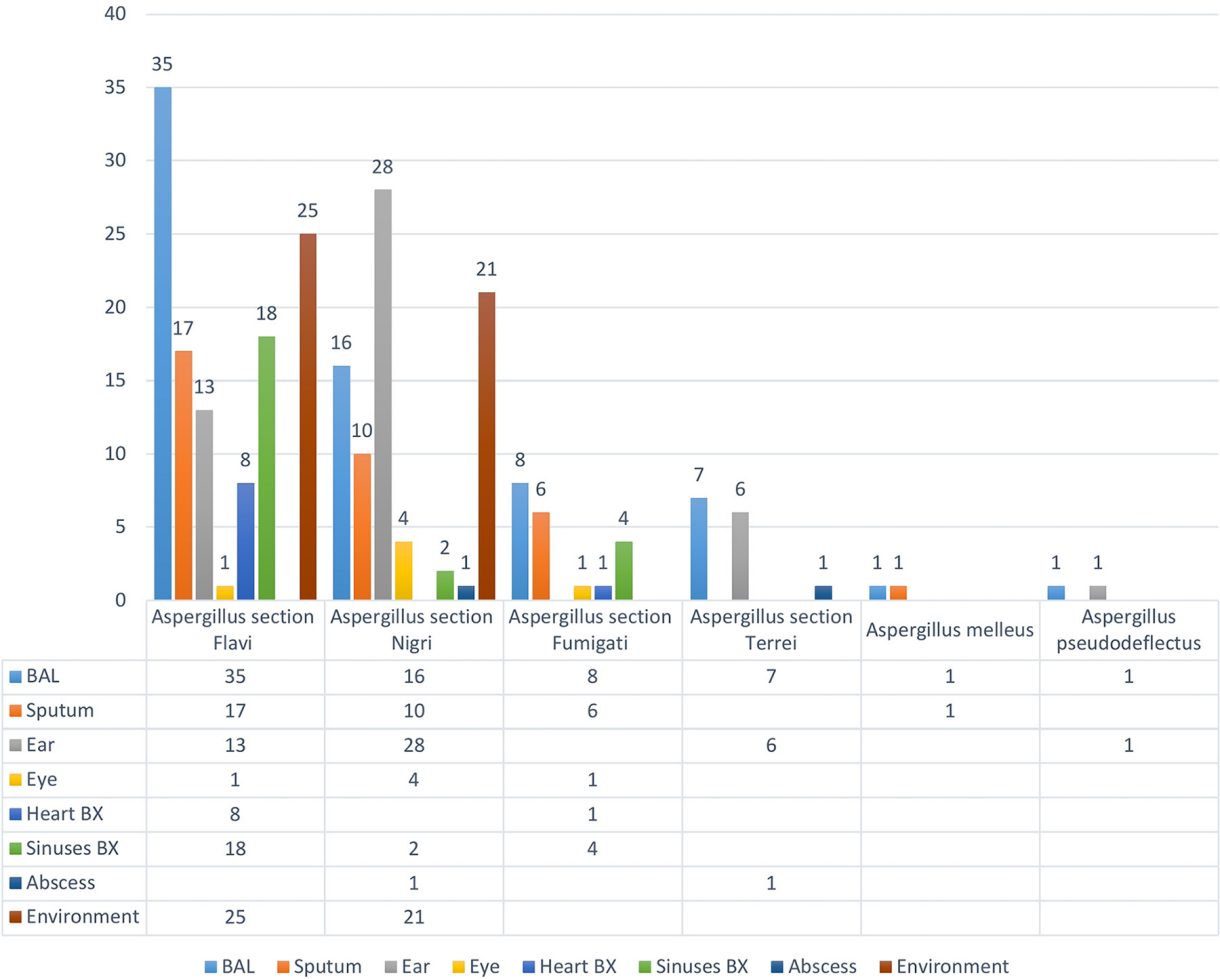
Site distribution of Aspergillus species isolates tested against seven antifungal agents. BAL, bronchoalveolar lavage (fluid); BX, biopsy specimen. The *y* axis shows the number of isolates.

Aspergillus section *Flavi* (117, 50.2%) was reported as the most prevalent species complex, followed by Aspergillus section *Nigri* (77, 33.1%), Aspergillus section *Fumigati* (21, 9%), Aspergillus section *Terrei* (14, 6%), Aspergillus pseudodeflectus (2, 0.85%), and Aspergillus melleus (2, 0.85%) (Fig. 1). Isolates of Aspergillus section *Flavi* were the most frequently isolated among all clinical specimens (*P* < 0.05), except in the ear canal, where the number of isolates of Aspergillus section *Nigri* was significantly higher. Regarding clinical origin types, 29.2% of the Aspergillus species isolates (68/233) were recovered from bronchoalveolar lavage fluid, 20.6% (48/233 isolates) from the ear canal, 14.6% (34/233 isolates) from sputum, 10.3% (24/233 isolates) from sinus biopsy specimen, 3.8% (9/233 isolates) from heart tissue, 2.6% (6/233 isolates) from the eye, 0.9% (2/233 isolates) from abscess (liver and brain abscess), and 18% (42/233 isolates) from hospital wards (bed, ventilator, and monitor) (Fig. 2). The number of isolates from respiratory tract samples (sputum, 34 isolates; bronchoalveolar lavage fluid, 68 isolates; and sinus secretions, 24 isolates) was significantly higher than that from other clinical samples (*P* < 0.05).

The results of the antifungal susceptibility tests, i.e., the minimum effective concentration (MEC)/MIC_50_, MIC/MEC_90_, epidemiological cutoff values (ECVs), and MICGM (geometric mean) distributions of seven antifungal agents for 233 *Aspergillus* species isolates are summarized in Tables 1 and 2. The MIC values of the reference control species were within accepted limits. Totally, for all the isolates tested, the lowest MIC_90_ value was observed for luliconazole (LUL) (0.016 μg/mL), followed by caspofungin (CAS) (MEC_90_, 0.125 μg/mL) and ISA (0.25 μg/mL). The MIC_90_ values for VOR, AMB, and posaconazole (POS) were 1, 2, and 2 μg/mL, respectively. Eighty-five Aspergillus isolates (36.5%) presented MIC values of ≥2 μg/mL for itraconazole (ITR). The MIC value of LUL significantly correlated positively with that of VOR (*P* = 0.04). Moreover, the MIC value of POS was significantly positively correlated with those of ITR (*P* = 0.03) and ISA (*P* = 0.001). The ECVs for AMB and ITR in Aspergillus section *Flavi* were 8 μg/mL. The MIC_90_ values in Aspergillus section *Nigri* for LUL, CAS, ISA, AMB, VOR, POS, and ITR were 0.016, 0.064, 1, 0.5, 1, 4, and ≥8 μg/mL, respectively. The MIC_90_ values of Aspergillus section *Fumigati* isolates for CAS, LUL, ISA, AMB, VOR, POS, and ITR were 0.064, 0.032, 0.25, 1, 1, 4, and ≥8 μg/mL, respectively. Aspergillus section *Terrei* isolates presented MIC_90_ values of 0.016, 0.064, and 0.5 μg/mL for LUL, ISA, and VOR, respectively. In the present study, strains with high MIC values for 2 or 3 azole antifungal drugs were observed but pan-azole-resistant Aspergillus species were not found (Table 3).

**TABLE 1:** MIC/MEC range, MIC_50_, MIC_90_, MIC_GM_, and epidemiological cutoff value distributions of Aspergillus species for seven antifungals, according to CLSI protocol

Aspergillus species (no. of isolates, %)	Antifungal agent	MIC/MEC range (μg/mL)	MIC/MEC_50_ (μg/mL)	MIC/MEC_90_ (μg/mL)	MIC_GM_ (μg/mL)	ECV (μg/mL)	Wild type (%)	Non-wild type (%)
Aspergillus section *Flavi* (117, 50.2%)	Amphotericin B	0.064–8	0.5	2	0.818	8	100	0.00
	Caspofungin	0.016–8	0.016	0.064	0.033	1	97.5	2.5
	Voriconazole	0.032–8	0.25	0.5	0.334	1	96.6	3.4
	Itraconazole	0.016–8	0.25	2	0.310	8	100	0.00
	Posaconazole	0.016–8	0.25	2	0.412	4	99	1
	Luliconazole	0.008–0.5	0.008	0.016	0.012	0.064	95	5
	Isavuconazole	0.008–2	0.032	0.064	0.032	0.25	96.6	3.4

Aspergillus section *Nigri* (77, 33.1%)	Amphotericin B	0.032–4	0.25	0.5	0.281	ND^*b*^	ND	ND
	Caspofungin	0.016–4	0.032	0.064	0.034	ND	ND	ND
	Voriconazole	0.032–2	0.5	1	0.425	ND	ND	ND
	Itraconazole	0.016–8	4	8	2.693	ND	ND	ND
	Posaconazole	0.064–8	0.5	4	0.799	ND	ND	ND
	Luliconazole	0.008–0.016	0.008	0.016	0.009	ND	ND	ND
	Isavuconazole	0.008–4	0.064	1	0.111	ND	ND	ND

Aspergillus section *Fumigati* (21, 9%)	Amphotericin B	0.032–2	0.5	1	0.327	ND	ND	ND
	Caspofungin	0.016–0.064	0.016	0.064	0.025	ND	ND	ND
	Voriconazole	0.064–2	0.5	1	0.469	ND	ND	ND
	Itraconazole	0.032–8	4	8	0.879	ND	ND	ND
	Posaconazole	0.064–8	1	4	1.941	ND	ND	ND
	Luliconazole	0.008–4	0.008	0.032	0.019	ND	ND	ND
	Isavuconazole	0.008–0.5	0.064	0.25	0.072	ND	ND	ND

Aspergillus section *Terrei* (14, 6%)	Amphotericin B	1–4	1	2	1.640	ND	ND	ND
	Caspofungin	0.016–4	0.125	1	0.171	ND	ND	ND
	Voriconazole	0.25–0.5	0.5	0.5	0.410	ND	ND	ND
	Itraconazole	0.25–4	0.5	1	0.322	ND	ND	ND
	Posaconazole	0.032–2	0.25	2	0.743	ND	ND	ND
	Luliconazole	0.008–0.016	0.008	0.016	0.013	ND	ND	ND
	Isavuconazole	0.016–0.125	0.016	0.064	0.030	ND	ND	ND

Total^*a*^ (233, 100%)	Amphotericin B	0.016–8	0.5	2	0.536	4	97	3
	Caspofungin	0.016–8	0.016	0.125	0.037	0.5	95.3	4.7
	Voriconazole	0.032–8	0.25	1	0.374	2	98.7	1.3
	Itraconazole	0.016–8	0.5	8	0.793	8	100	0.00
	Posaconazole	0.016–8	0.5	2	0.538	4	97.4	2.6
	Luliconazole	0.008–4	0.008	0.016	0.012	0.5	98.7	1.3
	Isavuconazole	0.008–1	0.032	0.25	0.051	2	98.3	1.7

a Two Aspergillus *pseudodeflectus* and two Aspergillus *melleus* isolates were included in the total number of isolates.

b ND, not determined.

**TABLE 2:** Patterns of *in vitro* susceptibility of 202 Aspergillus species isolates to seven antifungal agents

Aspergillus species	Antifungal agents	No. of isolates with MIC (μg/mL):										
		0.008	0.016	0.032	0.064	0.125	0.25	0.5	1	2	4	≥8
Aspergillus section *Flavi*	Amphotericin B				2	3	7	44	39	10	5	7
	Caspofungin		59	34	18			3	1			2
	Voriconazole				3	16	46	42	6	1	1	2
	Itraconazole		1	4	18	31	15	20	8	6	6	8
	Posaconazole		1		9	26	20	23	17	10	10	1
	Luliconazole	89	19	2	3			4				
	Isavuconazole	20	19	44	21	6	3	1	2	1	1	

Aspergillus section *Nigri*	Amphotericin B				3	25	17	18	11	1	1	
	Caspofungin		35	24	11	1	2	1		2	1	
	Voriconazole			2	1	11	15	26	14	8		
	Itraconazole		1		1	1	15			2	7	4
	Posaconazole				3	2	12	20	19	8	10	3
	Luliconazole	63	10	2	2							1
	Isavuconazole	7	7	6	16	19	8	2	1	7	4	

Aspergillus section *Fumigati*	Amphotericin B			5			2	7	5	2		
	Caspofungin		11	6	4							
	Voriconazole				2		6	6	4	3		
	Itraconazole			3			2		2		2	11
	Posaconazole				3		4	2	3	2	5	2
	Luliconazole	13	3	3							2	
	Isavuconazole	4		5	2	2	5	3				

Aspergillus section *Terrei*	Amphotericin B								7	4	3	
	Caspofungin		5			2	3		2			2
	Voriconazole						4	10				
	Itraconazole						3	4	5		2	
	Posaconazole			2	2		5			5		
	Luliconazole	5	9									
	Isavuconazole		7	3	2	2						

Total^*a*^	Amphotericin B		1	7	6	28	26	71	62	17	9	7
	Caspofungin		112	64	32	3	7	4	3	2	1	4
	Voriconazole			2	7	29	71	84	25	12	1	2
	Itraconazole		2	8	19	32	35	25	17	10	25	60
	Posaconazole		2	2	17	28	41	47	39	26	25	6
	Luliconazole	173	41	7	5			4		1	2	
	Isavuconazole	34	33	58	42	29	16	6	3	8	4	

a Aspergillus pseudodeflectus and Aspergillus melleus were included in the total number of isolates.

**TABLE 3:** Aspergillus species with multiple increasing MIC values to azole antifungals^*a*^

Isolate no.	Etiology	MIC (μg/mL) of antifungal:				
		Voriconazole	Posaconazole	Itraconazole	Luliconazole	Isavuconazole
1	Aspergillus flavus	4	0.25	2	0.5	0.25
2	Aspergillus flavus	0.5	1	4	0.016	0.064
3	Aspergillus fumigatus	0.5	2	8	0.008	0.25
4	Aspergillus fumigatus	1	1	8	0.008	0.064
5	Aspergillus fumigatus	0.5	8	8	0.008	0.008
6	Aspergillus fumigatus	2	4	8	0.032	0.25
7	Aspergillus fumigatus	1	4	4	0.016	0.5
8	Aspergillus fumigatus	2	4	8	0.032	0.125
9	Aspergillus fumigatus	2	4	8	0.032	0.25
10	Aspergillus fumigatus	2	4	8	0.016	0.25
11	Aspergillus fumigatus	1	4	4	0.016	0.5
12	Aspergillus fumigatus	0.5	2	8	0.008	0.250
13	Aspergillus niger	0.5	2	8	0.008	0.032
14	Aspergillus niger	0.5	1	8	0.008	0.125
15	Aspergillus niger	1	1	8	0.008	0.125
16	Aspergillus niger	0.5	1	8	0.008	0.064
17	Aspergillus niger	0.5	1	8	0.008	0.125
18	Aspergillus niger	1	1	2	0.008	0.25
19	Aspergillus niger	0.5	1	8	0.008	0.125
20	Aspergillus niger	0.5	4	8	0.008	0.008
21	Aspergillus niger	0.5	1	4	0.008	0.125
22	Aspergillus niger	0.5	1	8	0.008	0.125
23	Aspergillus niger	1	1	8	0.008	0.125
24	Aspergillus niger	0.5	1	8	0.008	0.064
25	Aspergillus niger	0.5	1	8	0.008	0.125
26	Aspergillus niger	1	1	8	0.008	0.25
27	Aspergillus niger	1	2	8	0.008	0.25
28	Aspergillus niger	1	1	8	0.008	0.25
29	Aspergillus niger	1	2	8	0.008	0.25
30	Aspergillus niger	0.5	2	4	0.008	0.25

a Itraconazole MIC value of ≥2 μg/mL with voriconazole MIC of ≥2 μg/mL or posaconazole MIC of ≥0.5 μg/mL.

## DISCUSSION

Among filamentous fungi, Aspergillus species are the most important agents of opportunistic infections in humans. The epidemiological distribution of Aspergillus species in each region is associated with the temperature, wind conditions, and humidity (18).

About 90% of Iran is made up of arid regions with various desert areas. Most Aspergillus isolates from Iran belonged to Aspergillus section *Flavi*, but these were reported as the second most common section of Aspergillus species in the United States and Brazil (3, 19). Species of Aspergillus section *Fumigati* were reported as having the greatest regional prevalence of Aspergillus species in China and the north of Portugal (1, 20).

Aspergillus isolates with resistance to antifungal agents are a public health concern. The ECVs of species of Aspergillus section *Flavi* for POS, ITR, and VOR (21); ISA (22); and AMB (23) were reported as 0.25, 1, 1, 1, and 2 μg/mL, respectively. The MIC_GM_ (geometric mean) values for species of Aspergillus section *Flavi* were reported as 0.009 μg/mL for LUL, 0.10 μg/mL for POS, 0.16 μg/mL for ISA, 0.24 μg/mL for ITR, 0.27 μg/mL for VOR, and 1.8 μg/mL for AMB (24). Moslem and Mahmoudabadi reported a MIC range for LUL in A. flavus isolates of 0.00049 to 0.00781 μg/mL, and the ECV for VOR was ≤ 2 μg/mL (25). Furthermore, 50% of A. flavus clinical isolates were found to be resistant to CAS (25). The discrepancies between these data and the results of our study in Iran could be due to the management of antifungal drug use in each region.

Species of Aspergillus section *Nigri* were reported as the most frequently isolated pathogens from otomycosis, but they are less commonly found in clinical samples (26). In the present study, the lowest MIC_GM_ values observed in Aspergillus section *Nigri* were for LUL, CAS, and ISA. According to the work of Hivary et al., the lowest MIC range, MIC_90_, and MIC_GM_ values for species of Aspergillus section *Nigri* in Iran belonged to LUL, and 86.7% of clinical isolates were sensitive to VOR (26). Moreover, 86.5%, 54.1%, and 83.8% of isolates in the work of Hivary et al. were found to be resistant to AMB, CAS, and POS, respectively (26), and these data are similar to those of the present study.

Species of Aspergillus section *Fumigati* are more pathogenic than other Aspergillus species and responsible for many fatal cases of aspergillosis (27, 28). According to the work of Mohammadi et al., the prevalence of azole-resistant isolates of Aspergillus section *Fumigati* increased from 3.3% to 6.6% in Iran (29). Nabili et al. found that the MIC_GM_ values of clinical isolates of Aspergillus section *Fumigati* for POS, CAS, VOR, ITR, and AMB were 0.049, 0.062, 0.085, 0.520, and 0.567 μg/mL, respectively (30). Aspergillus section *Terrei* is the cause of invasive and noninvasive mold infections in immunocompromised patients (31). Species of this section are characterized by intrinsic resistance to AMB and, sometimes, moderate susceptibility to azole antifungals (31). In the present study, the MIC_90_ value for AMB (2 μg/mL) of isolates from Aspergillus section *Terrei* was similar to those in a previous report from Iran (32). Six out of seven isolates of Aspergillus section *Terrei* from western China displayed low MICs for POS, ITR, VOR, and ISA (1). In another study by Zoran and coworkers, 5.4% of Aspergillus section *Terrei* isolates showed resistance to POS using the EUCAST method (33). The lowest MIC_GM_ of 40 environmental Aspergillus section *Terrei* strains from Iran was reported for LUL (0.00236 μg/mL), followed by POS (0.18621 μg/mL), VOR (0.22925 μg/mL), CAS (0.86 μg/mL), and AMB (11.12 μg/mL) (34). MIC_GM_ values for AMB and CAS were found to be lower in the present study than in a previous study (27). According to our data, LUL had excellent *in vitro* activity against isolates of Aspergillus section *Terrei* species, followed by CAS and ISA. The sensitivities of etiologic agents were correlated with the number of antifungal agents used for prophylaxis or treatment of the patients. Therefore, our data were in line with those from a study by Vaezi and coworkers (32). But, they also have differences from other studies due to the different management of health systems in different regions.

In recent years, azole cross-resistance in Aspergillus species has been reported (29–31). According to clinical data, resistance occurred during or after treatment with azole antifungals (28). Identification of azole-resistant Aspergillus species is an important aspect of clinical practice because azoles are a choice for the treatment of many fungal infections and can be used orally. Isavuconazole and LUL are new triazoles with *in vitro* activities against many fungal species (35), but they are not used in the clinics in our region. Due to a significant correlation with other azole antifungal agents, species with high LUL MIC values were seen in the present study. All Aspergillus species in the work of Schwarz and Dannaoui (30 isolates) exhibited MIC values for ISA ranging from 0.25 to 16 μg/mL, with MIC_50_, MIC_90_, and MIC_GM_ values of 1, 16, and 2.06 μg/mL, respectively (36). In addition, the ISA MIC value ranges for isolates of Aspergillus section *Flavi*, Aspergillus section *Fumigati*, Aspergillus nidulans, Aspergillus section *Nigri*, and Aspergillus section *Terrei* were reported as 2 to 4, 1 to 16, 0.25 to 0.5, 4 to 16, and 0.5 to 1 μg/mL, respectively (36). Buil et al. classified 221/487 isolates of A. fumigatus as ISA resistant based on the EUCAST breakpoint of 1 mg/L (37). Moreover, ISA MICs showed a high correlation with VOR MIC values but moderate and low correlations with ITR and POS MIC values, respectively (36). Howard et al. reported that 65% of 34 ITR-resistant species were also resistant to VOR and 74% (25/34) were cross-resistant to POS (28). Five (3.3 %) strains of A. fumigatus species analyzed by Nabili et al. showed cross-resistance to ITR, VOR, and POS (30). Moslem and Mahmoudabadi reported the resistance to two different classes of antifungals, AMB and CAS, for 15 cases (25). Similar to other studies, Aspergillus species with high MIC values for two or three azole antifungal agents were observed in the present study. However, pan-azole-resistant Aspergillus species were not observed. Simultaneous resistance to an azole, AMB, and CAS was not observed. Therefore, performing susceptibility testing can help physicians to properly treat aspergillosis. The limitation of the present study was the small number of isolates obtained for some species complexes. If the number of isolated species was above 100, we could have calculated wild-type (WT) and non-WT species.

According to our data, Aspergillus section *Flavi* species were the ones most commonly isolated from Iranian patients. Species of Aspergillus resistant to azoles, especially new antifungals, are worrisome. As a result, LUL, CAS, and ISA present high effective *in vitro* activity against Iranian isolates. Identifying the etiologic agents of Aspergillus infections and evaluating their susceptibility patterns can help efficient management of infection in high-risk patients.

## MATERIALS AND METHODS

In this cross-sectional study, Aspergillus species isolates from 11 medical university hospitals in Iran (i.e., Shiraz, Ahvaz, Isfahan, Kerman, Mashhad, Sanandaj, Sari, Tehran, Urmia, Yasuj, and Zahedan) were evaluated in the time period 2018 to 2021. Ethical approval was obtained from the ethics committee of the National Institute for Medical Research Development (IR. NIMAD. REC.1398.319).

### Sample collection and conventional and molecular identification.

Clinical samples (*n* = 3,500) from patients and environmental swabs from different wards of 11 Iranian hospitals were cultured on Sabouraud dextrose agar (SDA) plates (Merck, Germany) and incubated at room temperature for 7 to 10 days. The Aspergillus species were identified to the species complex level based on colony morphology, lactophenol cotton blue microscopy, PCR-restriction fragment length polymorphism (RFLP), and sequencing of the beta-tubulin gene. For DNA extraction, the isolated species were grown in Sabouraud dextrose broth (Merck, Darmstadt, Germany) for 2 to 3 days at 30°C and 120 rpm. DNA was extracted from young hyphae using the phenol-chloroform method. PCR amplification of the beta-tubulin gene was done using forward primer 5′-GGT AAC CAA ATC GGT GCT GCT TTC-3′ and reverse primer 5′-ACC CTC AGT GTG ACC CTT GGC-3′ (38). The PCR products were digested with a single AlwI restriction enzyme. Fifty isolated species were subjected to DNA sequencing. The data were analyzed with the NCBI nucleotide database (BLAST; https://blast.ncbi.nlm.nih.gov/Blast.cgi).

### Antifungal susceptibility testing.

Antifungal susceptibility tests of all the isolates were performed according to CLSI M38-A2 and M61 documents (15, 16). The seven antifungal agents involved in the present study were AMB, caspofungin (CAS), VOR, itraconazole (ITR), posaconazole (POS), luliconazole (LUL), and ISA (Sigma, UK). The antifungal agents were used at a final concentration of 8 to 0.016 μg/mL for AMB, CAS, VOR, ITR, and POS and 4 to 0.008 μg/mL for LUL and ISA. Briefly, Aspergillus isolates were grown on potato dextrose agar (Oxoid, England) at room temperature for 3 to 7 days. Spectrophotometrically, the turbidity of conidia was adjusted to optical densities between 0.09 and 0.11 at 530 nm and diluted at 1:50 in RPMI 1640 broth (Sigma-Aldrich, USA). Positive (antifungal-free) and negative (without fungus) controls were included on each row of the microdilution plate. The 96-well microdilution plates were incubated at 35°C and read after 24 and 48 h of incubation. The minimum concentration of CAS causing visible changes in morphological properties of the hyphae (round, compact, and branched hyphae) was defined as the minimum effective concentration (MEC) (15). The MIC endpoints for AMB and azole antifungal agents were the lowest concentration inhibiting visible fungal growth (100% inhibition), compared to growth of the controls. According to the CLSI M57 document (17), the epidemiological cutoff value (ECV) must be calculated from data extracted from ≥3 laboratories. In this study, the “eyeball” method was used for calculating ECV for Aspergillus section *Flavi* and total Aspergillus species (more than 100 individual data points). Candida krusei ATCC 6258 and Candida parapsilosis ATCC 22019 were used as quality controls in the same procedures.

### Statistical analysis.

The data were analyzed using SPSS software (version 16). The MIC/MEC ranges, MIC/MEC_50_ and MIC/MEC_90_, MIC geometric means (MIC_GM_), and ECVs were calculated for each Aspergillus species (17, 39). Correlations between the MIC values of the antifungal agents were evaluated by the Pearson correlation test and were significant at the 0.05 level.

### Data availability.

One Aspergillus pseudodeflectus (MZ668603), 16 Aspergillus niger (MZ668604 to MZ668619), one Aspergillus melleus (MZ668620), two Aspergillus flavus (MZ668621 and MZ668622), five Aspergillus luchuensis (MZ668623 to MZ668627) and 23 Aspergillus tubingensis (MZ668628 to MZ668650) isolate sequences were deposited in GenBank.
