# Erratum for Badiee et al., “Multicenter Study of Susceptibility of Aspergillus Species Isolated from Iranian University Hospitals to Seven Antifungal Agents”

**DOI:** 10.1128/spectrum.02255-23

**Published:** 2023-06-26

**Authors:** Parisa Badiee, Teun Boekhout, Ali Zarei Mahmoudabadi, Rasoul Mohammadi, Seyyed Amin Ayatollahi Mousavi, Mohammad Javad Najafzadeh, Jafar Soltani, Jamal Hashemi, Kambiz Diba, Abdolkarim Ghadimi-Moghadam, Ali Reza Salimi-Khorashad, Tahereh Shokohi, Maneli Amin Shahidi, Fatemeh Ghasemi, Hadis Jafarian

## ERRATUM

Volume 10, no. 3, e02539-21, 2022, https://doi.org/10.1128/Spectrum.02539-21. In the first paragraph of the Acknowledgments section, “award number IR.NIMAD.REC.1398.319” should read “award number 983657.”

